# Analysis of SAT Type Foot-And-Mouth Disease Virus Capsid Proteins and the Identification of Putative Amino Acid Residues Affecting Virus Stability

**DOI:** 10.1371/journal.pone.0061612

**Published:** 2013-05-22

**Authors:** Francois F. Maree, Belinda Blignaut, Tjaart A. P. de Beer, Elizabeth Rieder

**Affiliations:** 1 Transboundary Animal Diseases Programme, Onderstepoort Veterinary Institute, Agricultural Research Council, Onderstepoort, Pretoria, South Africa; 2 Department of Veterinary Tropical Diseases, Faculty of Veterinary Sciences, University of Pretoria, Onderstepoort, South Africa; 3 Department of Microbiology and Plant Pathology, Faculty of Agricultural and Natural Sciences, University of Pretoria, Pretoria, South Africa; 4 Bioinformatics and Computational Biology Unit, University of Pretoria, Pretoria, South Africa; 5 European Bioinformatics Institute, Wellcome Trust Campus, Hinxton, Cambridge, United Kingdom; 6 Foreign Animal Disease Research Unit, United States Department of Agriculture, Agricultural Research Service, Plum Island Animal Disease Center, Greenport, New York, United States of America; University of Kansas Medical Center, United States of America

## Abstract

Foot-and-mouth disease virus (FMDV) initiates infection by adhering to integrin receptors on target cells, followed by cell entry and disassembly of the virion through acidification within endosomes. Mild heating of the virions also leads to irreversible dissociation into pentamers, a characteristic linked to reduced vaccine efficacy. In this study, the structural stability of intra- and inter-serotype chimeric SAT2 and SAT3 virus particles to various conditions including low pH, mild temperatures or high ionic strength, was compared. Our results demonstrated that while both the SAT2 and SAT3 infectious capsids displayed different sensitivities in a series of low pH buffers, their stability profiles were comparable at high temperatures or high ionic strength conditions. Recombinant vSAT2 and intra-serotype chimeric viruses were used to map the amino acid differences in the capsid proteins of viruses with disparate low pH stabilities. Four His residues at the inter-pentamer interface were identified that change protonation states at pH 6.0. Of these, the H145 of VP3 appears to be involved in interactions with A141 in VP3 and K63 in VP2, and may be involved in orientating H142 of VP3 for interaction at the inter-pentamer interfaces.

## Introduction

Control of highly contagious diseases such as foot-and-mouth disease (FMD) by means of vaccination relies strongly on the chemical inactivation of complete viral particles. One of the principle factors which influences the potency of vaccine preparations and permits the induction of a protective antibody response, is the structural integrity of the intact virion typified by a sedimentation rate of 146S [1]. Other characteristics that play a role in vaccine efficacy include the productivity and replication efficiency of the vaccine strain in the production cell line, a close antigenic relationship to field viruses circulating in current outbreaks, and the ability to provide protection against a wide range of antigenic variants in the field [2], [3]. However, adaptation of new vaccine strains in the production cell line (BHK-21) is difficult and hampered by low amounts of stable antigen, often rendering this method inefficient for commercial vaccine manufacturing purposes [4]–[6]. In addition, the hot climate in sub-Saharan Africa, where the South African Territories (SAT) types are prevalent, also calls for vaccines with improved stability which are less reliant on a cold chain during storage and handling.

The aetiological agent, FMD virus (FMDV), is a single-stranded (ss) positive-sense RNA virus belonging to the genus *Aphthovirus* in the family *Picornaviridae* and exists as seven serotypes, *i.e.* A, C, O, SAT1, 2, 3, and Asia-1, with absence of cross protection [7]–[10]. Elucidation of the crystal structure of FMDV over 20 years ago [11] enabled assessment of the effects of acid and heat on the virus capsid structure and identification of protein-protein interactions that may correlate with enhanced acid or heat stability [12]–[14]. Reverse genetics combined with targeted mutagenesis of residues involved in the stability of the capsid is a powerful tool for the improvement of vaccines [15], [16], given that thermostability was linked to vaccine potency since the 1980s [1]. The self-assembly and stability of a multimeric protein capsid, such as FMDV, depends on the occurrence of numerous non-covalent interactions between multiple polypeptide subunits [11], [12], [14], [17]–[20]. The non-enveloped, icosahedral virion of FMDV is composed of 60 repetitions of four viral structural proteins, VP1-4. The three surface-exposed proteins, VP1, VP2 and VP3, assemble into a protomeric subunit, with the smaller VP4 located internally [9], [11], [12]. Subsequently, five protomers assemble into a pentameric intermediate and finally, 12 pentamers self-assemble through complex protein-protein interactions into a complete capsid [12]–[14], [21].

Even though FMDV, especially the SAT types, exhibit large intra- and inter-serotype genetic variability [22]–[25], the multiple and repetitive intersubunit interactions appear to have evolved under stringent and selective constraints [11], [13], [26], [27]. As a result the viral properties of each serotype are constrained within fundamental structural requirements of the virus capsid [26]–[29]. Viral capsid inter-subunit interactions are required to be sufficiently robust in order to provide stability to the capsid under environmental denaturing conditions [12], [13], whilst still permitting intracellular uncoating and release of viral RNA. Acidification of FMDV within cellular endosomes disrupts the interactions between VP2 and VP3 at the pentemeric interfaces leading to dissociation of the structure into pentamers, thereby releasing the viral RNA [30]–[34]. Mild heating of FMDV virions also leads to irreversible dissociation into pentamers, a characteristic associated with poor vaccine performance. This phenomenon, therefore, highlights the importance of a cold chain in the preservation of FMD vaccines [1], [13], [14].

The amino acid residues involved in protein-protein interactions at the pentameric interfaces and their intolerance to variation within serotype A and C viruses have previously been demonstrated [13], [14]. However, very little is known about residues located at the SAT pentameric interfaces involved in structural stability. Furthermore, by comparison to isolates from serotypes A, O and C, the SAT viral capsids are generally considered to be less stable [1]. Consequently, the principle aim of this study was to investigate the stability of SAT2 and SAT3 isolates in mildly acidic, heat or high ionic strength conditions. Interestingly, we showed that the SAT2 virions display similar acid lability compared to virions from serotypes A, O and C. In addition, our results demonstrate that amino acid residues at the pentameric interfaces may also contribute to acid stability of the SAT2 and SAT3 viruses. This is the first report that describes the identification of residues in the SAT virus capsids that may be replaced to allow the engineering of more stable capsids and consequently improved recombinant FMD vaccines.

## Materials and Methods

### Cells and viruses

Baby hamster kidney (BHK) clone 13 cells (strain 21; ATCC CCL-10) were maintained as described previously [35]. Virus stocks were prepared and titrated in BHK-21 cells using the plaque assay method [35]. Cultured BHK-21 cells were also used for RNA transfection and virus recovery. In addition, plaque assays were performed in either IB-RS-2 (Instituto Biologico renal suino) cells or Chinese hamster ovary (CHO) cells (strain K1; ATCC CCL-61), respectively propagated in RPMI medium (Sigma) and Ham's F-12 medium (Invitrogen) supplemented with 10% foetal calf serum (FCS, Delta Bioproducts). The GAG-deficient CHO derivative, pgsA-677 (CRL-2242), was also maintained in Ham's F-12 medium containing 10% FCS. COS-1 cells, transiently expressing the bovine integrin α_V_ subunit and either the β1, β3 or β6 subunits were used for cell binding studies [36], [37].

The wild-type SAT2 viruses used in this study were kindly provided by either the Transboundary Animal Diseases Programme (TADP, ARC-Onderstepoort Veterinary Institute, SA) or the FMD World Reference Laboratory at the Institute for Animal Health (IAH, Pirbright, UK). These viruses were subsequently either isolated on primary pig kidney (PK) or bovine thyroid (BTY) cells as part of the strategic stock maintenance and the passage history of the isolates have been described previously [16]. The viruses selected for this study included (1) three SAT2 viruses isolated from buffalo which originated from western Zimbabwe (SAT2/ZIM/7/83, SAT2/ZIM/17/91 and SAT2/ZIM/14/90); (2) a SAT2 virus from cattle outbreak in Zambia, (SAT2/ZAM/7/96); (3) a SAT3 virus isolated from buffalo originating in Zambia, (SAT3/ZAM/4/96). The passage history, host, country of origin and topotype are summarised in Table 1. For serial passages, infected or transfected 35-mm BHK-21 cell monolayers were frozen and thawed, and 1/10^th^ of the volume was used to inoculate a fresh BHK-21 monolayer. Following virus adsorption (with periodical rocking for 60 min at 37°C), virus growth medium (VGM; Eagle's basal medium (BME) with 1% FCS, 1% HEPES and antibiotics) was added, and the culture was incubated for no longer than 48 h at 37°C, after which the infected cells were frozen for subsequent passaging of the viruses.

**Table 1:** Summary of the different virus strains used in this study and their passage history.

FMDV strain	Host	Passage history	Country of origin	Topotype^a^	Chimeric virus^b^ and amino acid differences^c^
SAT2/ZIM/7/83	Bovine	B1BHK5B1	Zimbabwe	II (SAT2)	**vSAT2;** Y1169H, E1181A, L1182V
SAT2/ZIM/14/90	Buffalo	BTY1RS3	southern Zimbabwe	II (SAT2)	**vSAT2^ZIM14^SAT2;** V2015L, N3067I, S3087I, T3182A, K1174N
SAT2/ZIM/17/91	Buffalo	BTY2RS4	southern Zimbabwe	II (SAT2)	**vSAT2^ZIM17^SAT2;** V2015L, A2077T, C2164G, C1134G, E1179K
SAT2/ZAM/7/96	Buffalo	BTY1RS2	Zambia	III (SAT2)	**vSAT2^ZAM7^SAT2;** G2020R, E2214K, E1083K,
SAT3/ZAM/4/96	Buffalo	BTY1RS1	Zambia	4 (SAT3)	**vSAT3^ZAM4^SAT2**

The amino acid differences between the 1B/C/D-2A chimeric viruses and the parental isolates are indicated.

a Topotypes refers to genotypes distributed to specific geographic regions. The topotypes for the SAT serotypes were previously described [24], [25].

b Viruses recovered by transfection of BHK-21 cells with chimeric plasmids are designated “v” followed by the parental isolate number and the SAT2 plasmid used for cloning purposes. The amino acid residues have been numbered independently for each protein. For each residue, the first digit indicates the protein (VP1, VP2 or VP3) and the last three digits the amino acid position.

c The amino acid differences within the 1B/C/D-2A region of chimeric viruses and the parental viruses are indicated next to each respective viral protein.

### Titration and kinetics of virus production

Titrations were performed at least in duplicate in standard plaque assays by infecting monolayer cells in 35 mm cell culture plates (Nunc^TM^) with the respective viruses for 1 h, followed by the addition of a 2 ml tragacanth overlay [35]. Following incubation at 37°C for 28 or 40 h the overlayed infected monolayers were stained with 1% (w/v) methylene blue in 10% ethanol and 10% formaldehyde in phosphate buffered saline, pH 7.4.

One-step growth kinetic analyses were carried out in BHK-21 cells. Briefly, BHK-21 cells were infected with the virus strain for 1 h at a m.o.i. of 2–4 pfu/cell, washed with MBS-buffer (25 mM morpholine-ethanesulfonic acid, 145 mM NaCl, pH 5.5). Following incubation at 37°C for the indicated time intervals, the infected cells were harvested at 2, 4, 6, 8, 10, 12, 16 and 20 h post-infection (p.i.) and subsequently frozen at −70°C. Virus titers were determined and expressed as plaque forming units per millilitre (pfu/ml).

Monolayers of CHO-K1 or CHO-677 cells in 35 mm cell culture plates (Nunc^TM^) were infected with an m.o.i. of 5–10 PFU/ml of the parental and recombinant viruses. After 1 h of adsorption, cells were washed with MBS-buffer and then incubated with virus growth medium (VGM; Ham's F-12 with 1% FCS) at 37°C for 1 h and 24 h for each CHO cell type and frozen at −70°C. Virus titers were determined in BHK-21 cells and viral growth was calculated by subtracting the 1 h titer results from the 24 h titer results. Positive titers were interpreted as an indication that the viruses were able to infect and replicate in the CHO cells.

### RNA extraction, cDNA synthesis and construction of infectious, chimera cDNA clones

RNA was extracted from infected cell lysates with TRIzol® reagent (InVitrogen) according to the manufacturer's specifications and used as template for cDNA synthesis. Viral cDNA was synthesised with SuperScript III^TM^ (InVitrogen). The *ca*. 2.2 kb outer capsid-coding region of the SAT2 or SAT3 field isolates was obtained by PCR amplification. Unique *SspI* and *XmaI* sites were introduced at the 5′ and 3′ termini of the amplicons, respectively, to facilitate cloning into pSAT2 plasmid [15]. Briefly, the corresponding 2.2 kb fragment was excised from pSAT2 by digestion with *SspI* and *XmaI* restriction enzymes and the remaining fragment was ligated to the SAT2 and SAT3-specific amplicons. The resultant chimeric constructs, *i.e.* pSAT2^ZIM17^-SAT2, pSAT2^ZIM14^-SAT2, pSAT2^ZAM7^-SAT2 and pSAT3^ZAM4^-SAT2, were verified by sequencing using genome-specific oligonucleotides and the ABI PRISM^TM^ BigDye Terminator Cycle Sequencing Ready Reaction Kit v3.0 (Applied Biosystems).

### *In vitro* RNA synthesis, transfection and virus recovery

RNA was synthesized from *SwaI*-linearised plasmid DNA templates with the MEGAscript^TM^ T7 kit (Ambion). The transcript RNAs were examined by agarose gel electrophoresis to evaluate their integrity and the RNA concentrations were determined spectrometrically. BHK-21 cell monolayers, in 35-mm cell culture wells (Nunc^TM^), were transfected with the *in vitro*-generated RNA using Lipofectamine2000^TM^ (InVitrogen). The transfection medium was removed after 3–5 h and replaced with VGM, followed by incubation at 37°C for up to 48 h with a 5% CO_2_ influx. After one freeze-thaw cycle, the transfection supernatants were used for serial passaging on BHK-21 cells. BHK-21 monolayers in 35-mm cell culture wells were infected using 1/10^th^ of clarified infected supernatants and incubated for 48 h at 37°C. Viruses were subsequently harvested from infected cells by a freeze-thaw cycle and passaged four times on BHK-21 cells, using 10% of the supernatant from the previous passage. The recombinant viruses derived from the infectious chimeric cDNA clones were designated vSAT2^ZIM14^-SAT2, vSAT2^ZIM17^-SAT2, vSAT2^ZAM7^-SAT2 and vSAT3^ZAM4^-SAT2. Following the recovery of viable viruses, the presence of the inserts was verified once again with automated sequencing. Unless otherwise stated, viruses that were passaged four times were used for analysis.

### Cell-binding assay

Cell-binding studies were essentially performed as described previously [36], [37]. This entails the infection of COS-1 cells, transiently expressing the bovine integrin α_V_ subunit and either the β1, β3 or β6 subunits, with the vSAT2, pSAT2^ZIM17^-SAT2 and pSAT2^ZIM14^-SAT2 viruses, respectively. Sixteen hours after infection, cells were labelled with [^35^S]methionine and viral protein synthesis analyzed by radio-immunoprecipitation (RIP) of equal amounts of trichloroacetic acid-precipitable counts per minute using a SAT2 polyclonal serum followed by sodium dodecyl sulphate-polyacrylamide gel electrophoresis (SDS-PAGE). Radio-labelled proteins from non-transfected COS-1 cells and BHK-21 cells infected with vSAT2 were included as controls.

### Virus Neutralization test

The antigenic cross-reactivity of the SAT2/ZIM/7/83, SAT2/ZIM/17/91, SAT2/ZIM/14/90, SAT2/ZAM/7/96, vSAT2, pSAT2^ZIM17^-SAT2, pSAT2^ZIM14^-SAT2 and pSAT2^ZAM7^-SAT2viruses was determined using the micro-neutralization test essentially as described in the OIE Manual of Standards [38]. Reference cattle sera were prepared by two consecutive vaccinations (vaccinated on day 0, boosted on day 28 and bled on day 38) with the SAT2/ZIM/7/83, SAT2/KNP/19/89 and SAT2/ERI/12/98 vaccines. Cattle were housed in the isolation facility at TADP and all procedures were approved by the Onderstepoort Veterinary Institute Animal Ethics Committee and were performed according to national and international guidelines. IB-RS-2 cells were used as the indicator system in the neutralization test. The end point titer of the serum against homologous (SAT2/ZIM/7/83) and heterologous (SAT2/KNP/19/89 and SAT2/ERI/12/98) viruses was calculated as the reciprocal of the last dilution of serum to neutralise 100 TCID_50_ virus in 50% of the wells [39]. One-way antigenic relationships (r_1_-values) of the field isolates and engineered viruses relative to the reference sera were calculated and expressed as the ratio between the heterologous/homologous serum titer. All neutralization titer determinations were repeated at least twice and presented as an average.

### Sucrose density gradient purification

Culture fluids were harvested, clarified by centrifugation, concentrated with 8% PEG (w/v) and resolved on 10–50% (w/v) sucrose density gradients (SDG) by rate zonal centrifugation at 36,000 g for 16 h at 4°C. The gradients were fractionated and analysed spectrophotometrically by measuring the absorbancy at 260 nm. Fractions containing 146S virions were calculated using the extinction coefficient E_259nm_  = 79.9 [40] and pooled for analysis. The presence of the outer capsid proteins were verified using SDS-PAGE analysis, while the integrity of the RNA was verified by RT-PCR and sequencing of the 1D-coding region.

### Capsid dissociation assays and measurements of rate constants

The wild-type and recombinant SAT virus particles present in the cell culture supernatants or SDG purified samples were prepared in TNE buffer (100 mM Tris pH 7.4, 10 mM EDTA, 150 mM NaCl) essentially as described [41]. Briefly, 10^6^ to 10^7^ pfu/ml of infectious particles were mixed with TNE buffer ranging from pH 5.6 to 9.0 (±0.02) at a ratio of 1∶50 respectively. The mixtures were subsequently incubated for 30 min at room temperature. As a control, virus particles were also mixed with VGM at the same ratio as above. The samples were subsequently neutralised with 1 M Tris (pH 7.4), 150 mM NaCl and titrated on BHK-21 cells. Similarly, virus particles were treated for 30 min with TNE buffers with a constant pH of 7.4, containing NaCl concentrations ranging from 0.05–1.5 M (1∶50 virus particles to buffer), followed by titrations on BHK-21 cells. Alternatively the virus particles in TNE buffer (pH 7.4) were treated at temperatures of 25°C, 37°C, 45°C or 55°C for 30 minutes, after which the samples were cooled on ice and titrated. The 1∶50 dilution of the SDG purified particles ensured that the stabilising effect of sucrose was negligible as it was calculated the viscosity was less than 1%. All assays were performed in duplicate and the average virus titers were determined.

In addition, SDG-purified particles with an approximate titer of 4–8×10^6^ pfu/ml were either treated at pH 6.0, or heated at 42°C for different time intervals following a 1∶50 dilution in the appropriate TNE buffer. The number of infectious particles remaining after treatment was determined by plaque titrations on BHK-21 cells. The respective logarithmic values of the virus titers at the different time points (0, 15, 30, 45, 60, 90, 120 and 180 min p.i) were linearly fitted and the slopes were determined [42], [43] using the R statistical software [44]. The percentage of remaining infectious particles was also calculated and plotted along with the exponential decline used to calculate the inactivation rate constant as described [14].

### Structural analysis of variable amino acids in capsid subunits

Three-dimensional models of protomers comprising the four capsid proteins (1A, 1B, 1C and 1D) of the SAT2 viruses were constructed based on the crystallographic coordinates of O1BFS (1FOD) [45], while pentameric models were built using the capsid coordinates, A_10_61 (1ZBE). The models were based on an optimal amino acid alignment of the capsid proteins. Sequence alignments were performed with ClustalX software [46] using the default parameter setting. The modelling scripts were generated using the structural module in the FunGIMS software pakage and models were built using the Modeller 9v1 programme [47]. The homology structure was calculated by the satisfaction of spatial restraints as described by empirical databases. Structures were visualised with PyMol v0.98 (DeLano Scientific LLC). A PROPKA [48] analysis of each protomer was carried out to identify major protonation states affected by a pH of 6.0. Yasara [49] was used to analyse any hydrogen bond networks that were present.

pH-dependent differences between pentamers were investigated using a molecular dynamics simulation for ∼2.5 ns [49]. The simulation was performed at a pH of 6.0, water density of 0.997 g/ml and a NaCl concentration of 0.9% using the Amber99 force-field with periodic boundary conditions at a temperature of 298 K. A molecule consisting of two protomers (henceforth called the dimer) was also generated to analyse the interface between two pentamers.

## Results

### Construction and *in vitro* characterization of genetically engineered FMDV

**Figure 1:**
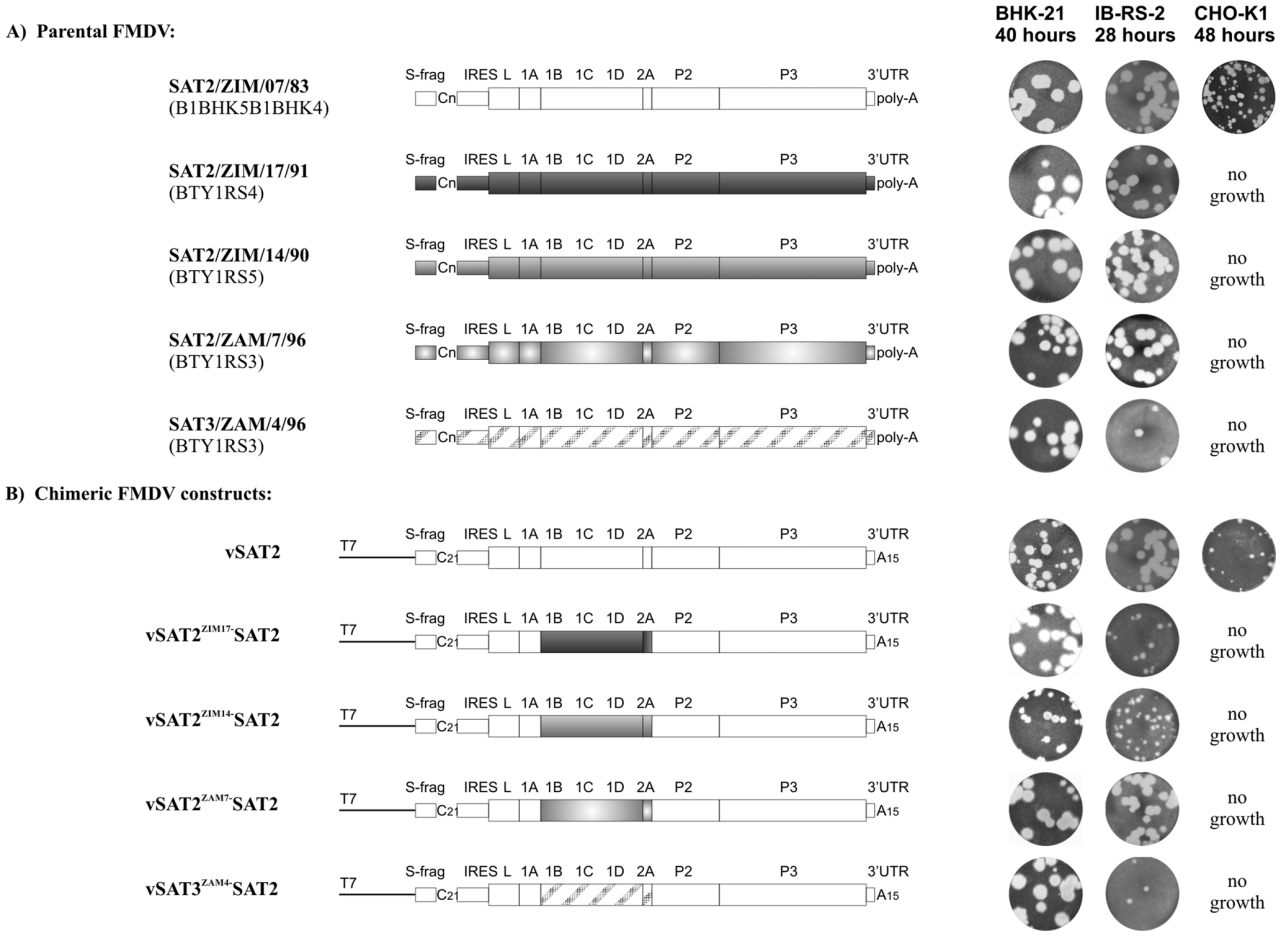
Schematic representation of (A) the genomes of the parental field viruses and (B) the chimeric FMDV constructs described in this study. Plaque morphologies of the parental and genetically engineered viruses obtained on monolayers of BHK-21, IB-RS-2 and CHO-K1 cells are shown. Cells infected with the respective viral strains were incubated for either 40 h (BHK-21 and CHO-K1) or 28 h (IB-RS-2).

To study the stability of SAT2 and SAT3 virions and the amino acid differences responsible for altered stabilities in a defined genetic background, we constructed infectious chimeric cDNA clones, containing the 1B/C/D/2A-coding region of SAT2 and SAT3 field isolates in the defined genetic background of an infectious pSAT2 genome-length clone (ZIM/7/83) [15]. Clones containing the capsid-coding region of southern Africa SAT2 isolates, *i.e.* SAT2/ZIM14/90, SAT2/ZIM/17/91 and SAT2/ZAM/7/96, as well as a SAT3 virus, SAT3/ZAM/4/96, were selected to synthesize RNA. The transcript RNAs were transfected into BHK-21 cells and viable chimeric viruses recovered. The recombinant viruses derived from the infectious chimeric cDNA clones were designated vSAT2^ZIM14^-SAT2, vSAT2^ZIM17^-SAT2, vSAT2^ZAM7^-SAT2 and vSAT3^ZAM4^-SAT2, respectively (Fig. 1). The vSAT3^ZAM4^-SAT2 is the only inter-serotype chimera containing the SAT3 outer capsid-coding region in a SAT2 genetic background.

Plaque morphologies, growth and antigenic properties of the recombinant viruses were examined to determine whether they resembled those of the parental viruses. As illustrated in Fig. 1, SAT2 and SAT3 field viruses, and the derived chimeric viruses produced large plaques (7–8 mm) on BHK-21 cells. The SAT2/ZIM/7/83 parental virus (passage history: B1BHK5B1) produced large plaques while its genetically-cloned derivative, vSAT2 (passage history: BHK3), produced a mixture of large and small (3–5 mm) plaques on BHK-21 cells. A porcine kidney cell line (IB-RS-2), known to express α_V_β8 [50], was also included in the analysis. The distribution of large plaques (7–8 mm) formed by the parental SAT2 and SAT3 viruses and vSAT2^ZAM7^-SAT2 on IB-RS-2 cells were similar to those observed on BHK-21 cells (Fig. 1). The vSAT2^ZIM14^-SAT2, vSAT2^ZIM17^-SAT2 and vSAT3^ZAM4^-SAT2 viruses formed medium plaques (3–5 mm) on IB-RS-2 cells (Fig. 1). In CHO-K1 (wild-type, glycosaminoglycan or GAG positive) cells, only the SAT2 vaccine strain, ZIM/7/83 and its derivative, vSAT2, were able to propagate, yielding small plaques (<2 mm) 48 h post-infection (p.i.) (Fig. 1). SAT2 and SAT3 field viruses, as well as their genetically-cloned derivatives, vSAT2^ZIM14^-SAT2, vSAT2^ZIM17^-SAT2, vSAT2^ZAM7^-SAT2 and vSAT3^ZAM4^-SAT2, were unable to produce plaques on CHO-K1 cells (Fig. 1). Subsequently, CHO-677 (GAG-deficient cell line) were also infected with the respective recombinant SAT viruses, but none of these viruses were able to replicate (not shown).

**Figure 2:**
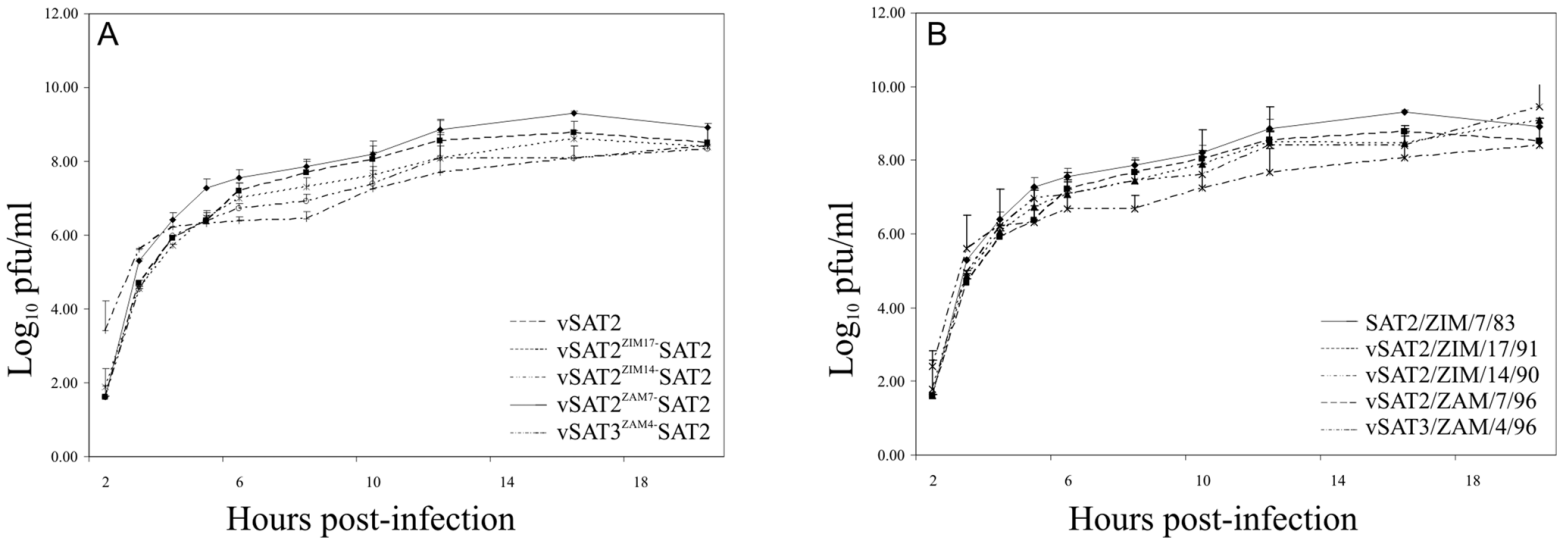
One-step growth kinetic studies were performed in BHK-21 cells. The average log virus titers are shown at different times p.i. with the vSAT2, vSAT2^ZIM17^-SAT2, vSAT2^ZIM14^-SAT2, vSAT2^ZAM7^-SAT2 and vSAT3^ZAM4^-SAT2 viruses (A) and the parental SAT2 and SAT3 viruses (B). For comparison of the relative release of virus particles from cells infected with the parental and chimeric viruses, the average virus titers were determined at different times p.i (2, 4, 6, 8, 10, 12, 16 and 20 h). The standard deviations of the titers determined from quadruple wells are indicated on the graph.

The growth kinetics of both field SAT2 and SAT3 and genetically-engineered intra- and inter-serotype viruses illustrates that the growth of the chimeric viruses, vSAT2^ZIM14^-SAT2, vSAT2^ZIM17^-SAT2, vSAT2^ZAM7^-SAT2 and vSAT3^ZAM4^-SAT2, were similar to that of the vSAT2 (Fig. 2A) and parental field viruses (Fig. 2B). Following infection of BHK-21 cells at a m.o.i. of 2–5 pfu/cell all viruses yielded high and comparable titers at 20 h p.i., after which their growth became indistinguishable.

Next we compared the antigenic profiles of the parental and the three intra-serotype chimeric viruses using SAT2 antisera from prototype strains in a virus neutralization (VN) assay (Table 2). The antigenic variation of the parental SAT2 isolates with the antisera was pronounced, returning r_1_-values of lower than 0.2, even though SAT2/ZIM/7/83, SAT2/ZIM/14/90 and SAT2/ZIM/17/91 belonged to the same topotype [25]. Based on the VN assay results, the r_1_-values of the chimeric viruses (Table 2) indicated that the sera reacted similarly to the recombinant SAT2 viruses, suggesting the absence of significant alterations in the structure of antigenic determinants on the chimeric virions. The r_1_-values of SAT2/ZAM/7/96 and vSAT2^ZAM7^-SAT2 against the reference sera were below 0.2. Hence, the results from the plaque morphology, growth kinetics and VN assays demonstrate that the immunological characteristics and receptor preferences of the parental field isolates were transferred to the chimeric viruses.

**Table 2:** Comparison of the antigenicity of SAT2 parental and chimeric viruses as measured against reference SAT2 antisera.

Viruses		r-values^a^	
		SAT2 test antisera^b^	
	anti-KNP/19/89^c^	anti-ZIM/7/83	anti-ERI/12/89^d^
SAT2/KNP/19/89^c^	**1.00**	0.17±0.05	0.27±0.09
SAT2/ZIM/7/83	0.28±0.03	**1.00**	0.25±0.04
SAT2/ZIM/17/91	0.09±0.03	0.13±0.02	0.17±0.05
SAT2/ZIM/14/90	0.16±0.03	0.07±0.02	0.15±0.05
SAT2/ZAM/7/96	0.09±0.04	0.12±0.03	0.18±0.05
SAT3/ZAM/4/96^e^	-	-	-
vSAT2^ZIM17^-SAT2	0.13±0.06	0.11±0.02	0.06±0.03
vSAT2^ZIM14^-SAT2	0.06±0.01	0.08±0.04	0.05±0.02
vSAT2^ZAM7^-SAT2	0.02±0.01	0.05±0.02	0.12±0.03
vSAT3^ZAM4^-SAT2^e^	-	-	-

a r-values are expressed as the ratio between the heterologous/homologous end point serum titers of the last dilution of serum to neutralize 100 TCID_50_ in 50% of the wells in VN assay. The average of two repeats is shown. The homologous r-values are in bold (n.d.  =  not done).

b The sera used in the VN assays were prepared by two consecutive vaccinations on day 0 and 28 and subsequently bled on day 38 with reference SAT2 viruses, i.e. SAT2/KNP/19/89, SAT2/ZIM/7/83 and SAT2/ERI/12/89.

c The SAT2/KNP/19/89 virus, belonging to SAT2 topotype 1 [25] and its homologous serum was included as a SAT2 control in the VN assay.

d SAT2/ERI/12/89 belong to the SAT2 topotype 12 [25] viruses from East Africa.

e VNT's were not performed against the SAT3 viruses using SAT2 antisera.

### Analysis of receptor usage by the engineered SAT type FMDV

**Figure 3:**
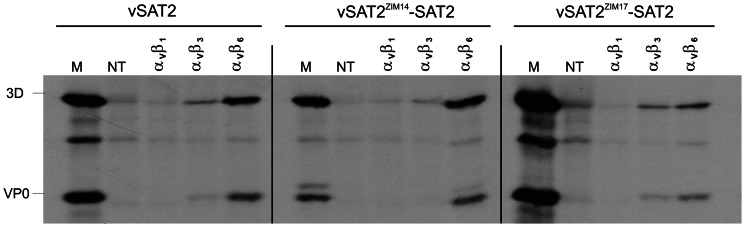
COS-1 cells were co-transfected with cDNA plasmids encoding the bovine integrin α_V_ subunit and either the β1, β3 or β6 subunits. Transfected cells were infected with the recombinant SAT2 viruses and proteins were radiolabelled with [^35^S]methionine. Viral protein synthesis were analyzed by radiolabelled immune-precipitation and SDS-PAGE. Immune-precipitated proteins from non-transfected but infected cell-lysates are indicated by “NT”, and the location of the viral structural proteins from lysates prepared from FMDV-infected BHK-21 cells is indicated by “M”.

The receptor specificity of the chimeric SAT2 viruses was investigated using COS-1 cells, co-transfected with bovine α_V_ integrin subunit and either of the β1, β3 or β6 subunit cDNAs. Replication of vSAT2 and the SAT2 chimeric viruses, vSAT2^ZIM14^-SAT2 and vSAT2^ZIM17^-SAT2, was demonstrated in cultured cells expressing α_V_β6 (Fig. 3). The bovine αvβ1 integrins were not able to sustain infection for any of these viruses. Only low level virus protein synthesis was detected in bovine αvβ3-expressing cells infected with vSAT2, vSAT2^ZIM14^-SAT2 and vSAT2^ZIM17^-SAT2.

### Stability of SAT viruses to biophysical conditions

**Figure 4:**
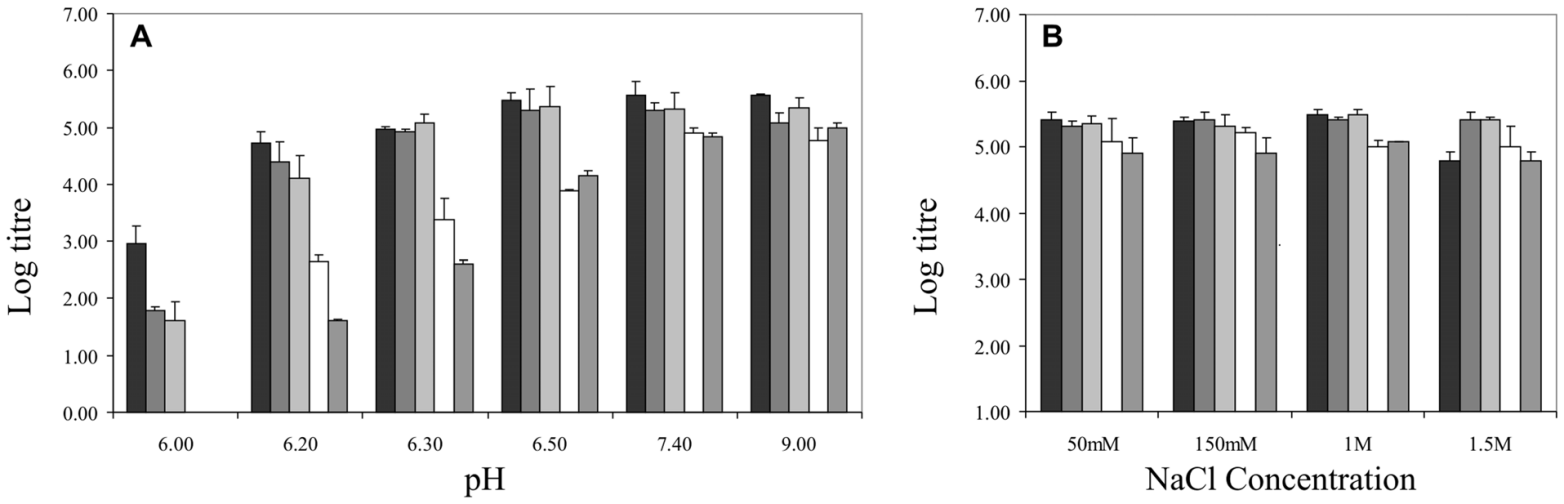
pH and ionic strength stability profiles of vSAT2 (black), vSAT2^ZIM14^-SAT2 (dark grey), vSAT2^ZIM17^-SAT2 (light grey), vSAT2^ZAM7^-SAT2 (white) and vSAT3^ZAM4^-SAT2 (medium grey). (A) Inactivation of SDG-purified virus particles following treatment with TNE (100 mM Tris, 150 mM NaCl, 10 mM EDTA) buffers ranging from pH 9.0 to 6.0 for 30 min. The average virus titers of two inactivation experiments at each pH are plotted. (B) The viruses were incubated in buffers with varying NaCl concentrations and the average log titers of two experiments are plotted.

The stability of SAT2 and SAT3 chimeric viruses to different pH, ionic strength and temperature conditions was evaluated. Treatment of the SDG-purified SAT2 and SAT3 chimeric viruses to buffered solutions of varying pH revealed intra-serotype differences with respect to viral capsid stability in mild acidic pH (Fig. 4A). Both vSAT2^ZIM17^-SAT2 and vSAT2^ZIM14^-SAT2 chimeric infectious particles (western lineage) displayed a comparable decrease in titer when the pH of the buffers were reduced from 9.0 to 6.2, with 21–25% infectivity remaining after 30 minutes at pH 6.2 (Fig. 4A). This stability profile was comparable to vSAT2 where 23% infectivity remained at pH 6.2, with a sudden drop in titer at a pH <6.2 (Fig. 4A). For the chimeric SAT2 viruses, the pH_50_ values, defined as the pH where 50% of infectivity is measurable, were calculated as 6.46 and 6.48 for vSAT2^ZIM14^-SAT2 and vSAT2^ZIM17^-SAT2, respectively. The pH_50_ value vSAT2 was calculated to be 6.51.

Conversely, vSAT2^ZAM7^-SAT2 revealed a significantly higher sensitivity in buffers at and below pH 6.5 as evidenced by a 260-fold decrease in infectious particles (1.5% infectivity remaining) within 30 min of incubation at pH 6.2, compared to the virus titer measured at pH 7.4 (Fig. 4A). At pH 6.0, no vSAT2^ZAM7^-SAT2 infectious particles were detected, while vSAT2, vSAT2^ZIM14^-SAT2 and vSAT2^ZIM17^-SAT2 maintained a titer of 10^2^ pfu/ml (Fig. 4A). The SAT3 inter-serotype chimera, vSAT3^ZAM4^-SAT2, showed a >2000-fold drop in titer when the pH was lowered from 7.4 to 6.2 and it diminished rapidly with no infectious particles detected at pH 6.0 (Fig. 4A). The pH_50_ values for the infectious virus particles were calculated as 6.8 and 6.81 for vSAT2^ZAM7^-SAT2 and vSAT3^ZAM4^-SAT2, respectively.

The recombinant virions showed remarkable resilience in a series of buffers with increasing ionic strengths (0.05–1.5 M NaCl; Fig. 4B). Similar virus titers were observed for purified vSAT2, vSAT2^ZIM14^-SAT2, vSAT2^ZIM17^-SAT2 and vSAT2^ZAM7^-SAT2 infectious 146 S particles in buffered solutions containing varying NaCl concentrations of between 0.05–1.5 M (Fig. 4B). Although differences in titers were observed, it is not believed to carry any biological relevance, with the exception of vSAT2 incubated in 1.5 M NaCl. A significant drop in titer was observed for vSAT2 after 30 min incubation in 1.5 M NaCl. It has been reported for A24 Cruzeiro empty capsids that increasing ionic strength (50 to 250 mM) destabilizes the capsids [12]. Although we did not investigate the effect of ionic strength on the SAT2 empty capsids, the destabilizing effect on infectious particles was observed at ten times the physiological salt concentration (1.5 M NaCl).

When incubated at temperatures ranging from 20°C to 55°C, the vSAT2, and the chimeric viruses, displayed similar thermostability with 6–14% residual infectivity remaining after incubation at 45°C for 30 min (data not shown). No infectious particles were detected after incubation at 55°C for 30 min.

### Heat and pH inactivation of SAT2 and SAT3 viruses

**Figure 5:**
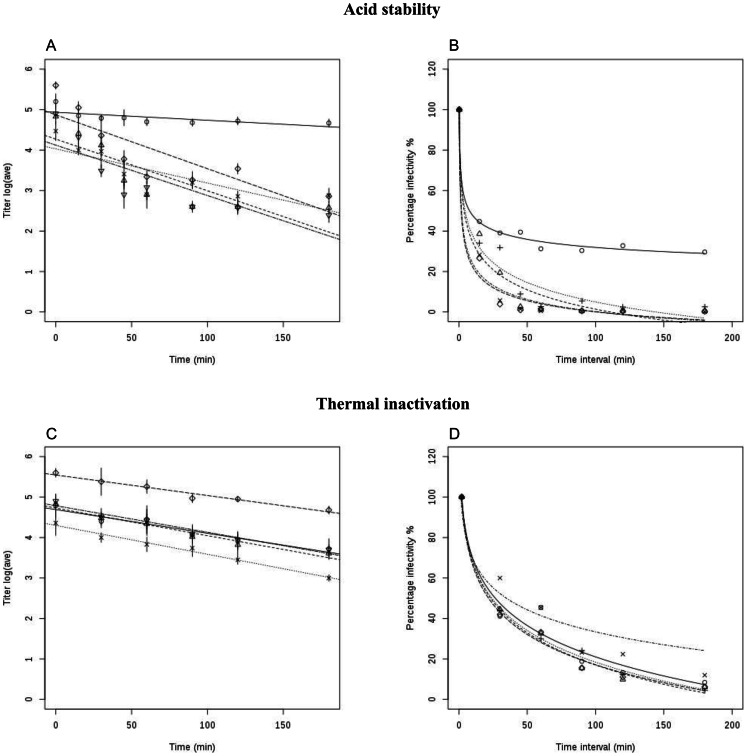
pH and thermal inactivation kinetics of vSAT2, vSAT2^ZIM17^-SAT2, vSAT2^ZIM14^-SAT2, vSAT2^ZAM7^-SAT2 and vSAT3^ZAM4^-SAT2 viruses. (A & C) Inactivation of SDG-purified vSAT2, vSAT2^ZIM14^-SAT2, vSAT2^ZIM17^-SAT2, vSAT2^ZAM7^-SAT2, and vSAT3^ZAM4^-SAT2 particles following treatment with TNE buffer at pH 6.0 (A) and heat treated at 42°C (C) for up to 3 h. The average log_10_ virus titers, as determined in two different inactivation experiments are shown. The respective logarithmic values of the virus titers at the different time points (0, 15, 30, 45, 60, 90, 120 and 180 min p.i.) were linearly fitted and the slopes were determined. (B & D) The average virus titers following pH treatment (B) or heat inactivation (D) were used to determine the percentage of residual infectivity over time.

The differences in the stability of the chimeric virions were further elucidated using pH and heat inactivation assays. These inactivation assays were done in a similar way, essentially SDG-purified chimeric particles at an approximate titer of 4–9×10^6^ pfu/ml, were either treated in a pH 6.0 buffer or at 42°C in a pH 7.4 buffer for different time intervals as indicated in Fig. 5. The observed inactivation profiles of the particles in a pH 6.0-buffered solution did not conform to a linear, but rather a logarithmic decrease in the number of infectious viral particles (Fig. 5A). When plotting the logarithmic titers over time, the acid lability of the chimeric viruses was reflected by the inactivation rate constant values at pH 6.0, which were 0.002, 0.008, 0.013, 0.013 and 0.012 per minute (min^−1^) for vSAT2, vSAT2^ZIM14^-SAT2, vSAT2^ZIM17^-SAT2, vSAT2^ZAM7^-SAT2 and vSAT3^ZAM4^-SAT2, respectively. The inactivation rates of vSAT2^ZIM17^-SAT2, vSAT2^ZAM7^-SAT2 and vSAT3^ZAM4^-SAT2 were higher than that of vSAT2 and vSAT2^ZIM14^-SAT2 viruses and were a reflection of the faster deterioration of the infectious particles following treatment in a pH 6.0 buffer (Fig. 5A). The vSAT2 virus showed the slowest rate of inactivation which resulted in *ca.* 24% of infectious particles remaining after 3 h treatment at pH 6.0 (Fig. 5B).

Contrary to the pH inactivation rates, no substantial differences with respect to thermostability were demonstrated between the vSAT2 and chimeric viruses (Fig. 5C). Temperature inactivation profiles can be drawn by plotting the decrease in virus logarithmic titers over time. The inactivation rate constants were then determined from the slope of the linear plots. The inactivation rate constant values at 42°C for vSAT2^ZAM7^-SAT2 and vSAT2 were 0.013 and 0.015 min^−1^, respectively, while the value for the remaining three chimeras was determined to be to be 0.018 min^−1^ (Fig. 5C). The decrease in the percentage of infectivity was similar to vSAT2 with 6–12% residual infectivity after 3 h at 42°C (Fig. 5D). Taken together, the results reveal that contrary to what we suspected, despite the variation in the outer capsid proteins the thermal stability of the FMDV particles was remarkably conserved.

### Molecular dynamics and the role of variable amino acids in the FMDV interpentameric interactions

**Figure 6:**
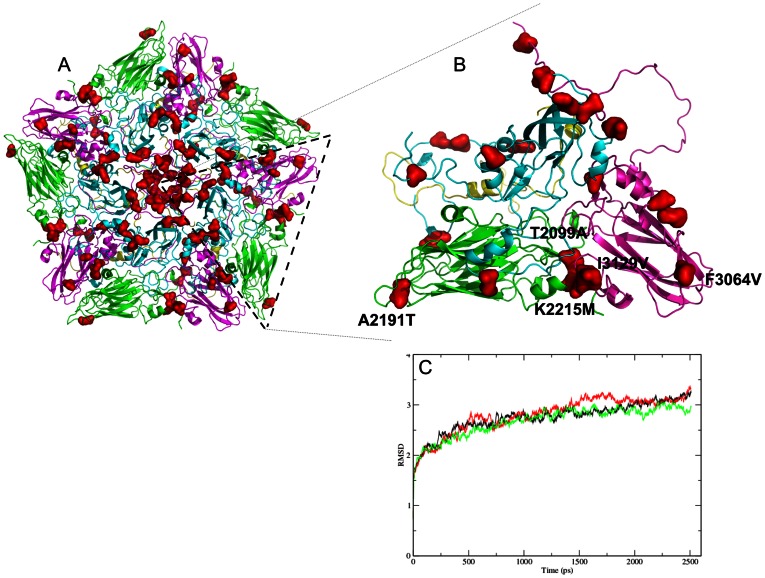
Variation observed in VP1-3 mapped to the structural models of the SAT2 (A) pentamers and (B) protomers. The amino alignment and effect of the variable residues is shown in Table S1. Residues predicted to play a role in the capsid stability are shown here. Variable positions are coloured in red, VP1 in cyan, VP2 in green, VP3 in magenta and VP4 in yellow. (C) The Cα RMSD variation of SAT2/ZIM/7/83 (in red), vSAT2 (in black) and vSAT2^ZAM7^-SAT2 (in green) over the ∼2.5 ns simulation time at pH 6.0 using the pentamers.

Structural mapping of variable amino acid residues within the outer capsid proteins of the chimeric SAT2 viruses that may play a role in the altered acid sensitivities was performed. The amino acid differences in the outer capsid proteins were mapped on the structure of a pentameric unit (Fig. 6) and their involvement in VP2–VP3 and VP2–VP2 pairwise contacts at the pentameric interface were analysed (Fig. S1, Table S1). The 50 amino acid differences that were observed between vSAT2 and the less acid-stable vSAT2^ZAM7^-SAT2 may suggest that some of these residues play a critical role in virion stability (Table S1).

Alignment of the capsid proteins showed that 10.5% of the residues were variable. The internally located 1A protein of the chimeric viruses and the vSAT2 virus shared 100% identity, due to the cloning strategy followed (Fig. 1). Although 1A may be involved in protein-RNA interaction or exert a small effect on inter-protomer interactions, the overall effect of 1A in the stability of the SAT2 virions was not taken into consideration in this analysis. The variation within the outer capsid proteins could be categorised into three groups, *i.e.* those having no effects (surface-exposed variable residues with no change in the local structure or interactions); those affecting the intra-protomer association; and those affecting the inter-protomer interactions (Fig. 6A).

The majority of the variable residues were surface-exposed, suggesting that these changes may exert a minimal effect on the stability of the capsid. At least 17 of the observed differences appeared to have putative effects on protomer-protomer interactions based on the predicted models (Fig. 6A and B). Although six of the variable residues could be considered as neutral and did not appear to influence the structure significantly, at least 11 of the 17 variable residues appeared to result in the addition or loss of interactions. Five of these residues mapped to the protomer particularly at the pentameric interface and adjacent to the pores located at the 3-fold axis (Fig. 6A and B). These differences may indeed influence the capsid assembly and disassembly dynamics, but still needs to be confirmed experimentally.

Molecular dynamics simulations were performed for ∼2.5 ns with the vSAT2, SAT2/ZIM/7/83 and vSAT2^ZAM7^-SAT2 pentamers. The root mean-square deviation (RMSD) variation over time for each of the pentamers at pH 6.0 is shown in Fig. 6C. Contrary to the lower acid stability observed for the purified vSAT2^ZAM7^-SAT2 infectious particles, there was no noteworthy difference in the RMSD of the pentamers of the viruses included in the study. Any significant difference between the infectious particles, such as pentamer dissociation, was expected to result in high RMSD values.

PROPKA analysis of the modelled FMDV protomer structures, however, indicated four His residues that play putative roles in capsid stability, *i.e.* H81 and H115 in VP2 and H145 and H172 in VP3. Of these, the His residue at position 145 of VP3 correlated with the residues at the pentameric interfaces that were previously identified and shown to contribute to virion stability [13]. These His residues are most likely involved in inter-pentamer interactions and are buried in the dimer interface, thereby concealing them from the aqueous phase. Changes in pKa values of the His residues in either a single protomer or two adjacent protomers (dimer) were determined with PROPKA analysis. The predicted pKa values of the protomers indicated that the four His residues changed protonation states in the region of pH 6.0. When the pattern of binding by these His residues was taken into consideration, it appeared as if none of the protomers of the SAT2 viruses, ZIM/7/83, vSAT2 or vSAT2^ZAM7^-SAT2, gained or lost a nett amount of bonds. However, when the residues were mapped to the predicted protein models, bond changes occurring between adjacent VP3 chains in the pentamer interfaces were clearly observed.

## Discussion

There is a large body of evidence suggesting that thermal stability of complete 146S FMDV particles and the immunogenicity of the particles could be linked to vaccine efficacy [1]. There is a common believe that SAT viruses are particularly unstable and very little is known about residues located at the SAT pentameric interfaces involved in structural stability. Consequently, we have investigated the biophysical stability of infectious virions generated from southern African isolates of the SAT2 and SAT3 serotypes under various controlled environmental conditions which are relevant during the vaccine production process.

We have demonstrated that chimeric viruses, containing the outer capsid of dissimilar viruses in a SAT2 genetic background, retained the plaque phenotypes, infectivity kinetics and immunological profiles of the parental strains (isolated during an outbreak) from which they were derived. With respect to cellular receptor preferences [50], [51], the SAT2 and SAT3 viruses originating from buffalo grew in BHK-21 or IB-RS2 cells following amplification in cell culture, but were unable to infect and replicate in CHO-K1 cells, suggesting that these field viruses do not utilize GAG receptors for cell entry. The interaction of SAT2 viruses with three α_V_β-integrin molecules was demonstrated in this study by expressing these integrin molecules transiently in COS cells. The results indicated that not all the integrins are used with the same affinity for cell entry by the SAT2 viruses. Whereas the SAT2 viruses were able to infect and replicate in COS cells expressing the α_V_β6 integrins, we found that these viruses displayed a poor ability to infect cells expressing α_V_β3 integrins, the result of which is in agreement with previous findings. with regards to the low affinity of type O_1_ viruses for α_V_β3 receptors [50], [52], [53]. This observation may be attributed to the G-H loop in VP1 that is structurally not optimal for binding to the α_V_β3 integrin [54]. Contrary to the ability of type O_1_ viruses to utilize α_V_β1 and α_V_β6 with a high efficiency [50], [52], [53], the α_V_β1 integrins were not able to mediate infection of any of the SAT2 viruses under the experimental conditions.

We found that virus particles of the SAT2 serotype, which differ by less than 11% in the capsid protein amino acid sequences, are stable in mild acidic conditions from pH 6.5 to 7.0. However, their infectivity was rapidly lost in buffers with a pH below 6.5, although infectious particles could still be detected at pH 6.0. The range of pH_50_ variation for the SAT2 serotype was 0.4 pH units between pH 6.8 and 6.4, with vSAT2^ZAM7^-SAT2 (SAT2/ZAM/7/96 derivative) being the least stable. The infectious vSAT3^ZAM4^-SAT2 particles also displayed a pronounced sensitivity under acidic conditions, losing 50% of its infectivity at pH 6.81. This variation in pH sensitivity did not appear to hamper the growth properties of the SAT2 or SAT3 viruses in cultured cells, an attribute to be expected of a virus that depends on acidification within endosomes for RNA release and replication [32].

SAT2 and SAT3 chimeric infectious particles were sensitive to mild heating. Thermal dynamics indicated that the SAT infectious particles decreased at a rate fitted best to single first order kinetics that is consistent with simple dissociation and similar to the behavior of a type C virus under mild temperature conditions [14]. It is known that mild heating of FMDV virions leads to irreversible dissociation into stable pentamers or 12S particles [1], [14]. Consequently, the first order kinetics observed for the SAT inactivation suggests similar dissociation of the infectious virions. The dissociation of virions in an acidic environment is considered to similarly lead to dissociation into pentamers and the observed inactivation rate was typified by a linear decrease of the logarithmic titer. Contrary to the thermal dynamics and acid lability of the SAT2 and SAT3 infectious particles, these particles were consistently stable in solutions with a high ionic strength and a pH as high as 9.0.

The stability of the FMD virion can be described as an equilibrium of multimeric electrostatic and hydrophobic interactions between the protein subunits and disruption of these interaction causes dissociation of the virion [11], [55], [56], [14]. Two likely residues, *i.e.* H142 and H143 in VP3, responsible for the destabilization of serotype A viruses as a function of pH, have been described previously [57], [58]. In an attempt to identify the relevant residues and side chain interaction that may cause the SAT2/ZAM/7/96 to be less stable in mild acidic conditions compared to the other SAT2 viruses, we mapped the amino acid variation to a modeled structure of the SAT2 capsid. While some of the amino acid variation was mapped to the surface of the virion, most of the variation was adjacent to the pore at the five fold axis of the virion (Table S1). These residues have the potential of interacting with VP4 or viral RNA at the inside of the pore [12]. When the variable surface-exposed residues were ignored on the basis that they may be involved in virus neutralisation or cell entry and, therefore, their effect on virion stability regarded to be minimal, at least 11 amino acid residues revealed potential protein-protein interaction. Five residues mapped to the pentamer interface, three (T2099A, K2215M and I3128V) of which shows interactions with residues at the C-terminus of the VP2 α-helix, responsible for interactions across the interface. A2191T and F3064V form hydrogen bonds across the interface at the 3-fold axis.

The pentamer models for vSAT2, SAT2/ZIM/7/83 and SAT2/ZAM/7/96 were built on the same template, therefore RMSD deviation after dynamic simulation could be directly compared. The molecular dynamics simulations of the related vSAT2 and SAT2/ZIM/7/83, and the distant SAT2/ZAM/7/96 showed that the pentamers were stable during the simulation as seen from the RMSD curves in Fig. 6. This indicates that the effect on the stability of the virus particles should be seen at the level of pentamer assembly into virions and not at a level of protomers assembling into pentamers. We also could not detect any change in the protonation state in any of the residue changes among the SAT2 viruses.

**Figure 7:**
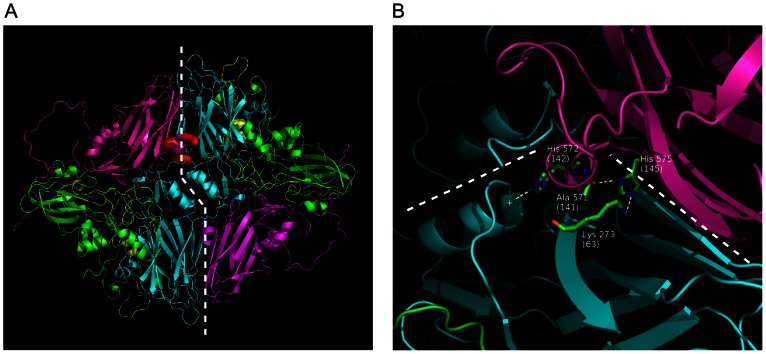
The interaction interface between two adjacent pentamers. (A) One protomer of each pentamer is shown. The dashed line indicates the interaction surface. VP3 residues H145, H142 and K63 using Van der Waals surfaces are coloured red. (B) The hydrogen bond network found in the pentamer interface. When VP3 H145 is neutral, it forms a hydrogen bond with VP2 K63 and VP3 A141. The neutral state seems to prevent pentamer association through H142 and H145. Yellow dashed lines indicate hydrogen bonds and the white dashed line indicates pentamer interface. The “+” indicates the charged dipole of the α-helix.

The pKa predictions results for the protomers indicated four His residues which change protonation states around pH 6.0. H145 in VP3 is involved in inter-protomer interaction on the pentameric interfaces and hydrogen bond analysis of the dimer molecule showed that this residue interacts with A141 in VP3 and with K63 in VP2 of the adjacent protomer (Fig. 7). The PROPKA results for the dimer molecule show the pKa for VP3 H145 to be -1.12. Thus, from these results it appears that a pH below 6.0 would disrupt interactions at the pentamer interface. A significant proportion of VP3 H145 needs to be neutral for pentamers to assemble into a capsid. This confirms previous observations that VP3 H145 plays a role in capsid disassembly and vaccine stability [12], [13], [57]. The VP3 H142 residue was also shown to be important in the association between the pentamers. The hydrogen bond analysis showed that VP3 H145 made a hydrogen bond with the backbone of VP3 A141 (Fig. 7B). This backbone hydrogen bond seems to be important is helping to orientate the VP3 H142 containing loop correctly to form the association with the charged dipole of the alpha-helix. The VP3 H145 also makes a hydrogen bond with VP2 K63 in the adjacent pentamer, thus providing extra interaction and stabilization between the pentamers (Fig. 7B). The loss of the hydrogen bonds with either VP2 K63 or VP3 A141 would have a significant effect on the interaction interface.

Our study provides evidence that SAT2 viruses isolated from buffalo enters the cell host with a preference for α_V_β6 integrin receptors. Moreover, the SAT virus particles were shown to dissociate within a range of mild acidic conditions which does not significantly impact on viral functions or infectivity. Furthermore, chimeric SAT viruses containing the immunological outer capsid-coding region of an emerging virus in the genetic background of a stable vaccine strain are infectious. Together, the data demonstrate the utility of recombinant DNA technology to produce chimeric viruses as vaccine seed stock with desirable properties and thermal-stability for the improvement of virus immunogenicity and vaccine efficacy.

## Supporting Information

Figure S1 Sequence alignment of the outer capsid proteins of the SAT2 viruses included in this study, i.e. ZIM/7/83, ZIM/14/90, ZIM/17/91 and ZAM/7/96.
(DOC)

Table S1 A summary of the variable amino acids in an alignment of the capsid proteins of the SAT2 viruses ZIM/7/83, ZIM/14/90, ZIM/17/91 and ZAM/7/96 and the possible interactions of the residues in the complete capsids.
(DOC)

## References

[1] Doel TR, Baccarini PJ (1981) Thermal stability of foot-and-mouth disease virus. Arch Virol 70: 21–32. 6277281 10.1007/BF01320790

[2] Barnett PV, Carabin H (2002) A review of emergency foot-and-mouth disease (FMD) vaccines. Vaccine 20: 1505–14. 11858856 10.1016/s0264-410x(01)00503-5

[3] Doel TR (2003) FMD vaccines. Virus Res 91: 81–99. 12527439 10.1016/s0168-1702(02)00261-7

[4] Pay TWF, Rweyemamu MM, O'Reilly KJ (1978) Experiences with Type SAT 2 foot-and-mouth disease vaccines in Southern Africa. XVth Conference of the Office International Des Epizzoties Permanent Commision on foot-and-mouth disease. p1–25.

[5] Preston KJ, Owens H, Mowat GN (1982) Sources of variations encountered during the selection and production of three strains of FMD virus for the development of vaccine for use in Nigeria. J Biol Stand 10: 35–45. 6279666 10.1016/s0092-1157(82)80046-2

[6] Amadori M, Berneri C, Archetti IL (1994) Immunogenicity of foot-and-mouth disease virus grown in BHK-21 suspension cells. Correlation with cell ploidy alterations and abnormal expression of the alpha 5 beta 1 integrin. Vaccine 12: 159–166. 7511862 10.1016/0264-410x(94)90055-8

[7] Bachrach HL (1968) Foot-and-mouth disease. Annu Rev Microbiol 22: 201–244. 4301615 10.1146/annurev.mi.22.100168.001221

[8] Pereira HG (1978) Antigenic variation in relation to epidemiology and control of foot and mouth disease. Br Vet J 35: 167–74.

[9] Sobrino F, Saiz M, Jiménez-Clavero MA, Núñez JI, Rosas MF, et al (2001) Foot-and-mouth disease virus: a long known virus, but a current threat. Vet Res 32: 1–30. 11254174 10.1051/vetres:2001106

[10] Domingo E, Baranowski E, Escarmís C, Sobrino F (2002) Foot-and-mouth disease virus. Comp Immunol Microbiol Infect Dis 25: 297–308. 12365806 10.1016/s0147-9571(02)00027-9

[11] Acharya R, Fry E, Stuart D, Fox G, Rowlands D, Brown F (1989) The three-dimensional structure of foot-and-mouth disease virus at 2.9 A resolution. Nature 337: 709–716. 2537470 10.1038/337709a0

[12] Curry S, Fry E, Blakemore W, Abu-Ghazaleh R, Jackson T, et al (1996) Perturbations in the surface structure of A22 Iraq foot-and-mouth disease virus accompanying coupled changes in host cell specificity and antigenicity. Structure 4: 135–145. 8805520 10.1016/s0969-2126(96)00017-2

[13] Ellard FM, Drew J, Blakemore WE, Stuart DI, King AM (1999) Evidence for the role of His-142 of protein 1C in the acid-induced disassembly of foot-and-mouth disease virus capsids. J Gen Virol 80: 1911–1918. 10466786 10.1099/0022-1317-80-8-1911

[14] Mateo R, Díaz A, Baranowski E, Mateu MG (2003) Complete alanine scanning of intersubunit interfaces in a foot-and-mouth disease virus capsid reveals critical contributions of many side chains to particle stability and viral function. J Biol Chem 278: 41019–41027. 12857761 10.1074/jbc.M304990200

[15] Van Rensburg HG, Henry T, Mason PW (2004) Studies of genetically defined chimeras of a European type A virus and a South African Territories type 2 virus reveal growth determinants for foot-and-mouth disease virus. J Gen Virol 85: 61–68. 14718620 10.1099/vir.0.19509-0

[16] Maree FF, Blignaut B, de Beer TAP, Visser N, Rieder E (2010) Mapping of amino acid residues responsible for adhesion of cell culture-adapted foot-and-mouth disease SAT type viruses. Virus Res 153: 82–91. 20637812 10.1016/j.virusres.2010.07.010

[17] Liljas L (1986) The structure of spherical viruses. Prog Biophys Mol Biol 48: 1–36. 3544053 10.1016/0079-6107(86)90008-8

[18] Rossmann MG, Johnson JE (1989) Icosahedral RNA virus structure. Annu Rev Biochem 58: 533–573. 2673017 10.1146/annurev.bi.58.070189.002533

[19] Reguera J, Carreira A, Riolobos L, Almendral JM, Mateu MG (2004) Role of interfacial amino acid residues in assembly, stability, and conformation of a spherical virus capsid. Proc Natl Acad Sci USA 101: 2724–2729. 14981262 10.1073/pnas.0307748101 PMC365688

[20] Reguera J, Grueso E, Carreira A, Sánchez-Martínez C, Almendral JM, et al (2005) Functional relevance of amino acid residues involved in interactions with ordered nucleic acid in a spherical virus. J Biol Chem 280: 17969–77. 15728575 10.1074/jbc.M500867200

[21] Mateo R, Luna E, Rincón V, Mateu MG (2008) Engineering viable foot-and-mouth disease viruses with increased thermostability as a step in the development of improved vaccines. J Virol 82: 12232–40. 18829763 10.1128/JVI.01553-08 PMC2593342

[22] Vosloo W, Kirkbride E, Bengis RG, Keet DF, Thomson GR (1995) Genome variation in the SAT types of foot-and-mouth disease viruses prevalent in buffalo (Syncerus caffer) in the Kruger National Park and other regions of southern Africa, 1986–93. Epidemiol Infect 114: 203–218. 7867739 10.1017/s0950268800052055 PMC2271348

[23] Van Rensburg HG, Nel LH (1999) Characterization of the structural-protein-coding region SAT 2 type foot-and-mouth disease virus. Virus Genes 19: 229–233. 10595414 10.1023/a:1008140815045

[24] Bastos ADS, Haydon DT, Forsberg R, Knowles NJ, Anderson EC, et al (2001) Genetic heterogeneity of SAT-1 type foot-an-d-mouth disease viruses in southern Africa. Arch Virol 146: 1537–1551. 11676416 10.1007/s007050170077

[25] Bastos ADS, Haydon DT, Sangare O, Boshoff CI, Edrich JL, et al (2003) The implications of virus diversity within the SAT 2 serotype for control of foot-and-mouth disease in sub-Saharan Africa. J Gen Virol 84: 1595–1606. 12771430 10.1099/vir.0.18859-0

[26] Mateu MG (1995) Antibody recognition of picornaviruses and escape from neutralization: a structural view. Virus Res 38: 1–24. 8546007 10.1016/0168-1702(95)00048-u

[27] Mateu MG, Valero ML, Andreu D, Domingo E (1996) Systematic replacement of amino acid residues within an Arg-Gly-Asp-containing loop of foot-and-mouth disease virus and effect on cell recognition. J Biol Chem 271: 12814–12819. 8662712 10.1074/jbc.271.22.12814

[28] Samuel AR, Knowles NJ (2001) Foot-and-mouth disease virus: cause of the recent crisis for the UK livestock industry. Trends Genet 17: 421–424. 11485797 10.1016/s0168-9525(01)02374-5

[29] Rossmann MG, Greve JM, Kolatkar PR, Olson NH, Smith TJ, et al.. (1997) in Structural Biology of Viruses (Chiu W, Garcea R, Burnette R, eds). 105–133, Oxford University, Press, Oxford.

[30] Brown F (1963) Cartwright (1963) Purification of radioactive foot-and-mouth disease virus. Nature 199: 1168–70. 14072035 10.1038/1991168a0

[31] Burroughs JN, Rowlands DJ, Sangar DV, Talbot P, Brown F (1971) Further evidence for multiple proteins in the foot-and-mouth disease virus particle. J Gen Virol 13: 73–84. 4108674 10.1099/0022-1317-13-1-73

[32] Carrillo EC, Giachetti C, Campos RH (1984) Effect of lysosomotropic agents on the foot-and-mouth disease virus replication. Virology 135: 542–545. 6330983 10.1016/0042-6822(84)90208-3

[33] Curry S, Fry E, Blakemore W, Abu-Ghazaleh R, Jackson T, et al (1997) Dissecting the roles of VP0 cleavage and RNA packaging in picornavirus capsid stabilization: the structure of empty capsids of foot-and-mouth disease virus. J Virol 71: 9743–9752. 9371640 10.1128/jvi.71.12.9743-9752.1997 PMC230284

[34] Van Vlijmen HWT, Curry S, Schaefer M, Karplus M (1998) Titration calculations of foot-and-mouth disease virus capsid and their stabilities as a function of pH. J Mol Biol 275: 295–308. 9466910 10.1006/jmbi.1997.1418

[35] Rieder E, Bunch T, Brown F, Mason PW (1993) Genetically engineered foot-and-mouth disease viruses with poly(C) tracts of two nucleotides are virulent in mice. J Virol 67: 5139–5145. 8394441 10.1128/jvi.67.9.5139-5145.1993 PMC237911

[36] Duque H, Baxt B (2003) FMDV receptors: comparison of bovine αv integrin utilization by type A and O Viruses. J Virol 77: 2500–2511. 12551988 10.1128/JVI.77.4.2500-2511.2003 PMC141088

[37] Duque H, LaRocco M, Golde WT, Baxt B (2004) Interactions of foot-and-mouth disease virus with soluble bovine αVβ3 and αVβ6 integrins. J Virol 78: 9773–81. 15331710 10.1128/JVI.78.18.9773-9781.2004 PMC514961

[38] Office International des Epizooties (2009). In Manual of Diagnostic Tests and Vaccines for Terrestrial Animals 2009, Chapter 2.1.5. 1–25. Office International des Epizooties, Paris, France.

[39] Rweyemamu MM, Ouldridge EJ, Head M, Ferrari R (1984) The effect of antiserum quality on strain specificity assessment of foot and mouth disease virus by the neutralization reaction. J Biol Stand 12: 295–303. 6090465 10.1016/s0092-1157(84)80009-8

[40] Doel TR, Mowat GN (1985) An international collaborative study on foot and mouth disease virus assay methods. 2. Quantification of 146S particles. J Biol Stand 13: 335–44. 2997228 10.1016/s0092-1157(85)80048-2

[41] Knipe T, Rieder E, Baxt B, Ward G, Mason P (1997) Characterization of synthetic foot-and-mouth disease virus provirions separates acid-mediated disassembly from infectivity. J Virol 71: 2851–2856. 9060641 10.1128/jvi.71.4.2851-2856.1997 PMC191410

[42] Mateo R, Mateu MG (2007) Deterministic, compensatory mutational events in the capsid of foot-and-mouth disease virus in response to the introduction of mutations found in viruses from persistent infections. J Virol 81: 1879–87. 17151123 10.1128/JVI.01899-06 PMC1797555

[43] Mateo R, Luna E, Mateu MG (2007) Thermostable variants are not generally represented in foot-and-mouth disease virus quasispecies. J Gen Virol 88: 859–864. 17325358 10.1099/vir.0.82521-0

[44] R Development Core Team (2009) R: A Language and Environment for Statistical Computing.

[45] Logan D, Abu-Ghazaleh R, Blakemore W, Curry S, Jackson T, et al (1993) Structure of a major immunogenic site on foot-and-mouth disease virus. Nature 362: 566–568. 8385272 10.1038/362566a0

[46] Thompson JD, Gibson TJ, Plewniak F, Jeanmougin F, Higgins DG (1997) The CLUSTAL_X windows interface: flexible strategies for multiple sequence alignment aided by quality analysis tools. Nucleic Acids Res 25: 4876–82. 9396791 10.1093/nar/25.24.4876 PMC147148

[47] Fiser A, Sali A (2003) Modeller: generation and refinement of homology-based protein structure models. Methods Enzymol 374: 461–491. 14696385 10.1016/S0076-6879(03)74020-8

[48] Li H, Robertson AD, Jensen JH (2005) Very fast empirical prediction and rationalization of protein pKa values. Proteins 61: 704–721. 16231289 10.1002/prot.20660

[49] Krieger E, Nielsen JE, Spronk CA, Vriend G (2006) Fast empirical pKa prediction by Ewald summation. J Mol Graph Model 25: 481–486. 16644253 10.1016/j.jmgm.2006.02.009

[50] Burman A, Clark S, Abrescia NG, Fry EE, Stuart DI, et al (2006) Specificity of the VP1 GH Loop of foot-and-mouth disease virus for αv integrins. J Virol 80: 9798–9810. 16973584 10.1128/JVI.00577-06 PMC1617245

[51] Jackson T, Clarck SJ, Berryman S, Burman A, Cambier S, et al (2004) Integrin αvβ8 functions as a receptor for foot-and-mouth disease virus: Role of the β-chain cytodomain in integrin-mediated infection. J Virol 78: 4533–4540. 15078934 10.1128/JVI.78.9.4533-4540.2004 PMC387692

[52] Jackson T, Mould AP, Sheppard D, King AMQ (2002) Integrin αvβ1 is a receptor for foot-and-mouth disease virus. J Virol 76: 935–941. 11773368 10.1128/JVI.76.3.935-941.2002 PMC135819

[53] Neff S, Mason PW, Baxt B (2000) High-efficiency utilization of the bovine integrin αvβ3 as a receptor for foot-and-mouth disease virus is dependent on the bovine β3 subunit. J Virol 74: 7298–7306. 10906183 10.1128/jvi.74.16.7298-7306.2000 PMC112250

[54] Boettiger D, Lynch L, Blystone S, Huber F (2001) Distinct ligand-binding modes for integrin α_v_β_3_-mediated adhesion to fibronectin versus vitronectin. J Biol Chem 276: 31684–90. 11423542 10.1074/jbc.M103997200

[55] Lea S, Hernandez J, Blakemore W, Brocchi E, Curry S, et al (1994) The structure and antigenicity of a type C foot-and-mouth disease virus. Structure 2: 123–139. 8081743 10.1016/s0969-2126(00)00014-9

[56] Lea S, Abu-Ghazaleh R, Blakemore W, Curry S, Fry E, et al (1995) Structural comparison of two strains of foot-and-mouth disease virus subtype O_1_ and a laboratory antigenic variant G67. Structure 3: 571–580. 8590018 10.1016/s0969-2126(01)00191-5

[57] Twomey T, France LL, Hassard S, Burrage TG, Newman JFE, et al (1995) Characterization of an acid-resistant mutant of foot-and-mouth disease virus. Virology 206: 69–75. 7831827 10.1016/s0042-6822(95)80020-4

[58] Curry S, Abrams CC, FryE, Crowther JC, Belsham GJ, et al (1995) Viral RNA modulates the acid sensitivity of Foot-and-mouth disease virus capsids. J Virol 69: 430–438. 7983739 10.1128/jvi.69.1.430-438.1995 PMC188591

