# Supplementary material for: Analysis of SAT Type Foot-And-Mouth Disease Virus Capsid Proteins and the Identification of Putative Amino Acid Residues Affecting Virus Stability
Source: PLoS One. 2013 May 22;8(5):e61612. doi: 10.1371/journal.pone.0061612 (PMC3661562; doi:10.1371/journal.pone.0061612)
Supplement: Figure S1 — Sequence alignment of the outer capsid proteins of the SAT2 viruses included in this study, i.e. ZIM/7/83, ZIM/14/90, ZIM/17/91 and ZAM/7/96. (DOC) [file pone.0061612.s001.doc]

Maree *et al.*, Stability of SAT FMDV

Feb 2013

**Supplementary data**

**VP2**

#ZIM/07/83 DKKTEETTLL EDRIVTTRHG TTTSTTQSSV GITYGYADAD SFRPGPNTSG LETRVEQAER FFKEKLFDWT SDKPFGTLYV LELPKDHKGI YGSLTDAYTY

#ZIM/14/90 .......... .......... .......... ........S. ...S...... .......... .......... .........I .......... .....ES.A.

#ZIM/17/91 .......... .......... .......... ........S. .......... .......... .......... ......A... .......... .....ES.A.

#ZAM/07/96 .......... .......... .......... .......... .......... .......... .......... .......... .......... ........A.

#ZIM/07/83 MRNGWDVQVS ATSTQFNGGS LLVAMVPELC SLKDREEFQL SLYPHQFINP RTNTTAHIQV PYLGVNRHDQ GKRHQAWSLV VMVLTPLTTE AQMQSGTVEV

#ZIM/14/90 .......... .......... .......... ..RA...... .......... .......... .......... .......... .......... ...N......

#ZIM/17/91 .......... .......... .......... ..R....... .......... .......... ...C...... .....T.... .......... ...N......

#ZAM/07/96 .........T .......... ....L..... ..RE...... T......... .......... .......... .......... .......... T..T......

**VP3**

#ZIM/07/83 YANIAPTNVF VAGEKPAKQG IIPVACFDGY GGFQNTDPKT ADPIYGYVYN PSRNDCHGRY SNLLDVAEAC PTFLNFDGKP YVVTKNNGDK VMTCFDVAFT

#ZIM/14/90 .......... ....M..... ......S... .......... .......... .......... .......... ....D..... .......... ..........

#ZIM/17/91 .......... .......... .V....S... .......... .......... .......... .......... .......... .......... ..........

#ZAM/07/96 .......... ....M..... .V....A... .......... .......... .......... .......... ..L....... .......... ..........

#ZIM/07/83 HKVHKNTFLA GLADYYAQYQ GSLNYHFMYT GPTHHKAKFM VAYIPPGIET DRLPKTPEDA AHCYHSEWDT GLNSQFTFAV PYVSASDFSY THTDTPAMAT

#ZIM/14/90 .....S.... ......T... .......... .......... .......TA. .K........ .......... .......... .......... ..........

#ZIM/17/91 .......... ......T... .......... .......... .......V.. .K........ .......... .......... .......... ..........

#ZAM/07/96 .......... ......T... .......... .......... .......V.. .K........ .......... .......... .......... ..........

**VP1**

#ZIM/07/83 TNGWVAVFQV TDTHSAEAAV VVSVSAGPDL EFRFPVDPVR QTTSSGEGAD VVTTDPSTHG GAVTEKKRVH TDVAFVMDRF THVLTNRTAF AVDLMDTNEK

#ZIM/14/90 .......Y.. .......... .......... .....I..I. .......... .......... .S.A..R.M. .......... ...H..K... ..........

#ZIM/17/91 .......Y.. .......... .......... .....I.... ......G... .......... .S.M..R.M. .......... ...H..K.S. VI........

#ZAM/07/96 .......Y.. .......... .......... .....I.... ....A..... .......... .R.V..R.M. ......L... ...H..K.T. N......K..

#ZIM/07/83 TLVGGLLRAA TYYFCDLEIA CLGEHERVWW QPNGAPRTTT LRDNPMVFSH NNVTRFAVPY TAPHRLLSTR YNGECKYTQQ STAIRGDRAV LAAKYANTKH

#ZIM/14/90 ....A....S .......... .I.D.K.... .........Q .......... .S.....L.. .......... .....N...R .P........ .......V..

#ZIM/17/91 ....A....S ........V. .I.T.K.... .........Q .......... .S.....L.. .......... ........ER A......W.. ..........

#ZAM/07/96 ....A....S .......... .V...A..Y. .........Q .G........ .K.....I.. .......A.. .........E AR........ ......GA..

#ZIM/07/83 KLPSTFNFGH VTADKPVDVY YRMKRAELYC PRPLLPGYDH ADRDRFDSPI GVEKQ

#ZIM/14/90 E........F .......... F....T.... ......A... G......A.. .....

#ZIM/17/91 E........F ....E..... ...E...... ......V... GN.....A.. .....

#ZAM/07/96 S......... ....AA.... .......... ......A.E. S......A.. .....

**Figure S1:** Sequence alignment of the outer capsid proteins of the SAT2 viruses included in this study, i.e. ZIM/7/83, ZIM/14/90, ZIM/17/91 and ZAM/7/96.
