# Supplementary material for: Analysis of SAT Type Foot-And-Mouth Disease Virus Capsid Proteins and the Identification of Putative Amino Acid Residues Affecting Virus Stability
Source: PLoS One. 2013 May 22;8(5):e61612. doi: 10.1371/journal.pone.0061612 (PMC3661562; doi:10.1371/journal.pone.0061612)
Supplement: Table S1 — A summary of the variable amino acids in an alignment of the capsid proteins of the SAT2 viruses ZIM/7/83, ZIM/14/90, ZIM/17/91 and ZAM/7/96 and the possible interactions of the residues in the complete capsids. (DOC) [file pone.0061612.s002.doc]

Maree *et al.*, Stability of SAT FMDV

Feb 2013

**Supplementary data**

**Table S1:** A summary of the variable amino acids in an alignment of the capsid proteins of the SAT2 viruses ZIM/7/83, ZIM/14/90, ZIM/17/91 and ZAM/7/96 and the possible interactions of the residues in the complete capsids.

| **Residue number** | **Protein** | **Amino acid** | | | | **Interaction** |
| --- | --- | --- | --- | --- | --- | --- |
|  |  | **ZIM/7/83**  **(vSAT2)** | **ZIM/14/90** | **ZIM/17/91** | **ZAM/7/96** |  |
| 2039  2044  2077  2080  2096  2097  2099  2110  2125  2133  2134  2141  2164  2176  2191  2194  2215  3003  3008  3054  3056  3064  3087  3098  3129  3130  3133  3189  3217  3220  1004  1021  1023  1026  1028  1036  1043  1046  1048  1050  1051  1057  1064  1069  1078  1081  1083  1085  1088  1099  1101  1111  1117  1127  1135  1139-41  1147  1156-7  1160  1169  1174  1175  1180  1183  1185  1196  1198  1200  1201  1207 | VP2  VP3  VP1 | A  P  T/M  V  D  A  T  S  M  K  D  S  G  A  A  Q  K  I  F  F  N  V/F  N  A  I  E  R  F  V  V  S  A  T  K  V  M  L  R  A  A  V  N  G  A  I  L  E  E  W  T  R  N  V  S  K  QST  R  NT  K  K  K  P  Y  K  A  G  D  A  D  S | S  S  T  I  E  S  A  S  M  R  A  S  G  A  A  N  M  I  S  F  D  V  S  T  T  A  K  Y  I  I  S  S  A  R  M  M  H  K  A  A  V  N  A  S  I  I  D  K  W  Q  R  S  L  S  N  RSP  R  NV  E  K  K  P  F  K  T  A  D  G  D  A | S  P  A  V  E  S  A  S  M  R  D  S  C  T  A  N  K  V  S  F  N  V  N  T  V  E  K  Y  I  V  S  S  M  R  M  M  H  K  S  V  I  N  A  S  V  I  T  K  W  Q  R  S  L  S  K  RAT  W  NT  E  E  E  P  Y  E  A  V  D  G  N  A | A  P  T  V  D  A  A  T  L  R  E  T  G  A  T  T  M  V  A  L  N  V  N  T  V  E  K  Y  I  V  A  R  V  R  M  L  H  K  T  N  V  K  A  S  I  V  E  A  Y  Q  G  K  I  A  K  EAR  R  GA  S  A  A  A  Y  K  A  A  E  S  D  A | No effect  No effect  P2074; intra-protomer  No effect  Ionic; inter-pentamer  Intra-protomer  P3127; intra-protomer  Y2201; K3120  No effect  Conserved charge  Inter-protomer  Intra-protomer  Intra-protomer  No effect  Inter-pentamer  Inter-pentamer  P3127; intra-protomer  Inter-protomer  Inter-protomer  Inter-protomer  Inter-protomer  Inter-protomer  Inter-protomer  Intra-protomer  Inter-protomer  Inter-protomer  Conserved charge  Hydrophobic  Intra-protomer  Intra-protomer  No effect  Inter-protomer  No effect  Conserved charge  No effect  Intra-protomer  No effect  No effect  No effect  Inter-protomer  No effect  Inter-protomer  No effect  No effect  No effect  No effect  No effect  No effect  Intra-protomer  No effect  Inter-protomer  Inter-protomer  No effect  No effect  No effect  No effect  No effect  No effect  No effect  No effect  No effect  No effect  No effect  No effect  No effect  No effect  Conserved charge  Inter-protomer  No effect  No effect |
